# Prediction of oxygen requirement in patients with COVID-19 using a pre-trained chest radiograph xAI model: efficient development of auditable risk prediction models via a fine-tuning approach

**DOI:** 10.1038/s41598-022-24721-5

**Published:** 2022-12-07

**Authors:** Joowon Chung, Doyun Kim, Jongmun Choi, Sehyo Yune, Kyoung Doo Song, Seonkyoung Kim, Michelle Chua, Marc D. Succi, John Conklin, Maria G. Figueiro Longo, Jeanne B. Ackman, Milena Petranovic, Michael H. Lev, Synho Do

**Affiliations:** 1 Department of Radiology, Massachusetts General Brigham and Harvard Medical School, Boston, MA USA; 2 Department of Radiology, Samsung Medical Center, Sungkyunkwan University School of Medicine, 81 Irwon-Ro, Gangnam-Gu, Seoul, 06351 Republic of Korea

**Keywords:** Diagnostic markers, Predictive markers, Biomarkers, Health care, Medical research, Engineering, Mathematics and computing

## Abstract

Risk prediction requires comprehensive integration of clinical information and concurrent radiological findings. We present an upgraded chest radiograph (CXR) explainable artificial intelligence (xAI) model, which was trained on 241,723 well-annotated CXRs obtained prior to the onset of the COVID-19 pandemic. Mean area under the receiver operating characteristic curve (AUROC) for detection of 20 radiographic features was 0.955 (95% CI 0.938–0.955) on PA view and 0.909 (95% CI 0.890–0.925) on AP view. Coexistent and correlated radiographic findings are displayed in an interpretation table, and calibrated classifier confidence is displayed on an AI scoreboard. Retrieval of similar feature patches and comparable CXRs from a Model-Derived Atlas provides justification for model predictions. To demonstrate the feasibility of a fine-tuning approach for efficient and scalable development of xAI risk prediction models, we applied our CXR xAI model, in combination with clinical information, to predict oxygen requirement in COVID-19 patients. Prediction accuracy for high flow oxygen (HFO) and mechanical ventilation (MV) was 0.953 and 0.934 at 24 h and 0.932 and 0.836 at 72 h from the time of emergency department (ED) admission, respectively. Our CXR xAI model is auditable and captures key pathophysiological manifestations of cardiorespiratory diseases and cardiothoracic comorbidities. This model can be efficiently and broadly applied via a fine-tuning approach to provide fully automated risk and outcome predictions in various clinical scenarios in real-world practice.

## Introduction

Comprehensive integration of clinical information and radiological findings is required for assessment of disease severity and risk prediction. Conventional chest radiography is inexpensive, routinely obtained in the emergency department (ED), and available in low resource settings. However, radiographic features have only been utilized for fully automated risk prediction in relatively few machine learning models^1–5^.

We considered various approaches to developing machine learning models for risk prediction. A full training approach, which involves end-to-end training from scratch for each prediction task, is disease-specific. However, convolutional neural networks (CNNs) for image analysis are computationally expensive to train and can easily over-fit to small training datasets. A significant amount of time, cost, and expertise is required for accurate annotation of large imaging datasets. This limits the scalability and efficiency of the full training approach.

In our proposed fine-tuning approach, an auditable explainable AI (xAI) model is pre-trained on a well-curated training dataset to detect clinically important radiological features and subsequently applied, in combination with disease-specific clinical data, to a variety of risk prediction tasks. As safety in medicine is paramount, it is crucial for prediction models to be auditable. This is an important consideration, as careful development of high quality xAI models will require time and resources.

We have recently introduced a method for automated labeling of chest radiograph (CXR) using a newly developed xAI model^6^. Prediction uncertainty for detection of pneumonia, pulmonary edema, atelectasis, pleural effusion, and cardiomegaly was quantified via (1) feature localization and construction of a patch feature Atlas for visual similarity assessment, and (2) computation of calibrated classifier confidence for each prediction.

In this study, we introduce an upgraded CXR xAI model, which can simultaneously detect 20 abnormal radiographic features. This CXR xAI model was trained on well-annotated CXRs obtained at a tertiary teaching hospital prior to the onset of the COVID-19 pandemic. Patch similarity and calibrated classifier confidence are utilized for auditability. In addition, an interpretation table displays coexistent and correlated radiographic findings as well as comparable CXRs extracted from the Model-Derived Atlas for similarity assessment. We further demonstrate the viability of a fine-tuning approach for efficient and scalable development of risk prediction models by using our CXR xAI model to predict oxygen requirement in patients with COVID-19.

As our CXR xAI model is auditable and captures important pathophysiological manifestations of cardiorespiratory diseases and cardiothoracic comorbidities, we believe that it can be broadly applied for risk prediction.

## Results

### Explainable AI (xAI) model development and performance

We developed a CXR xAI model to detect 20 radiographic features using well-annotated CXRs obtained at a tertiary teaching hospital prior to the onset of the COVID-19 pandemic. Mean AUROC for detection of the 20 radiographic features was 0.955 (95% CI 0.938–0.955) on the posteroanterior (PA) view and 0.909 (95% CI 0.890–0.925) on the anteroposterior (AP) view (Table 1). On the PA view, the achieved AUROC was greater than 0.950 for cardiomegaly, pulmonary edema, emphysema, pleural effusion, other pleural lesions, non-fracture bone abnormality, mediastinal abnormality, diaphragmatic abnormality, decreased lung volume, and increased lung volume. On the AP view, the achieved AUROC was greater than 0.950 for pleural effusion, other interstitial opacity, other pleural lesions, emphysema, and foreign body. On the PA view, the achieved AUROC was less than 0.9 for fracture and nodule/mass only. Supplementary Fig. 1 shows the receiver operating characteristic curves (ROCs) for each label, derived from the final model applied to the test set.

**Table 1:** AUROCs derived from application of the final model to the test set.

Ensemble (6 models) category	PA view AUROC (95% CI)	AP view AUROC (95% CI)
Fracture	0.850 (0.774–0.910)	0.721 (0.545–0.895)
Non-fracture	0.988 (0.975–0.998)	0.932 (0.839–0.997)
Diaphragm	0.966 (0.935–0.992)	0.885 (0.735–0.987)
Foreign body	0.948 (0.930–0.965)	0.965 (0.954–0.974)
Aorta	0.938 (0.920–0.955)	0.917 (0.891–0.941)
Cardiornegaly	0.965 (0.952–0.978)	0.917 (0.899–0.935)
Hilar area	0.944 (0.903–0.978)	0.872 (0.725–1.000)
Mediastinum	0.980 (0.954–0.999)	0.889 (0.783–0.968)
Cavity/cyst	0.936 (0.869–1.000)	0.891 (0.781–0.970)
Emphysema	0.998 (0.994–1.000)	0.986 (0.977–0.993)
Atelectasis	0.942 (0.925–0.958)	0.862 (0.840–0.884)
Nodule/mass	0.798 (0.709–0.882)	0.925 (0.874–0.967)
Other interstitial opacity	0.941 (0.888–0.984)	0.991 (0.982–0.998)
Pulmonary edema	0.978 (0.966–0.988)	0.915 (0.897–0.932)
Pneumonia	0.904 (0.875–0.930)	0.805 (0.771–0.838)
Decreased lung volume	0.976 (0.957–0.992)	0.912 (0.891–0.932)
Increased lung volume	0.977 (0.957–0.994)	NA
Other pleural lesions	0.980 (0.942–0.999)	0.977 (0.934–1.000)
Pleural effusion	0.982 (0.969–0.991)	0.951 (0.939–0.962)
Pneumothorax	0.950 (0.914–0.978)	0.864 (0.820–0.904)
Average score	0.947 (0.938–0.955)	0.909 (0.890–0.925)

Non-fracture refers to non-fracture bone abnormality. Diaphragm, Aorta, Hilar area, and Mediastinum refer to diaphragmatic abnormality, aortic abnormality, hilar abnormality, and mediastinal abnormality, respectively. As there were no cases with increased lung volume in the test set, the AUROC for this label could not be calculated.

**Figure 1:**
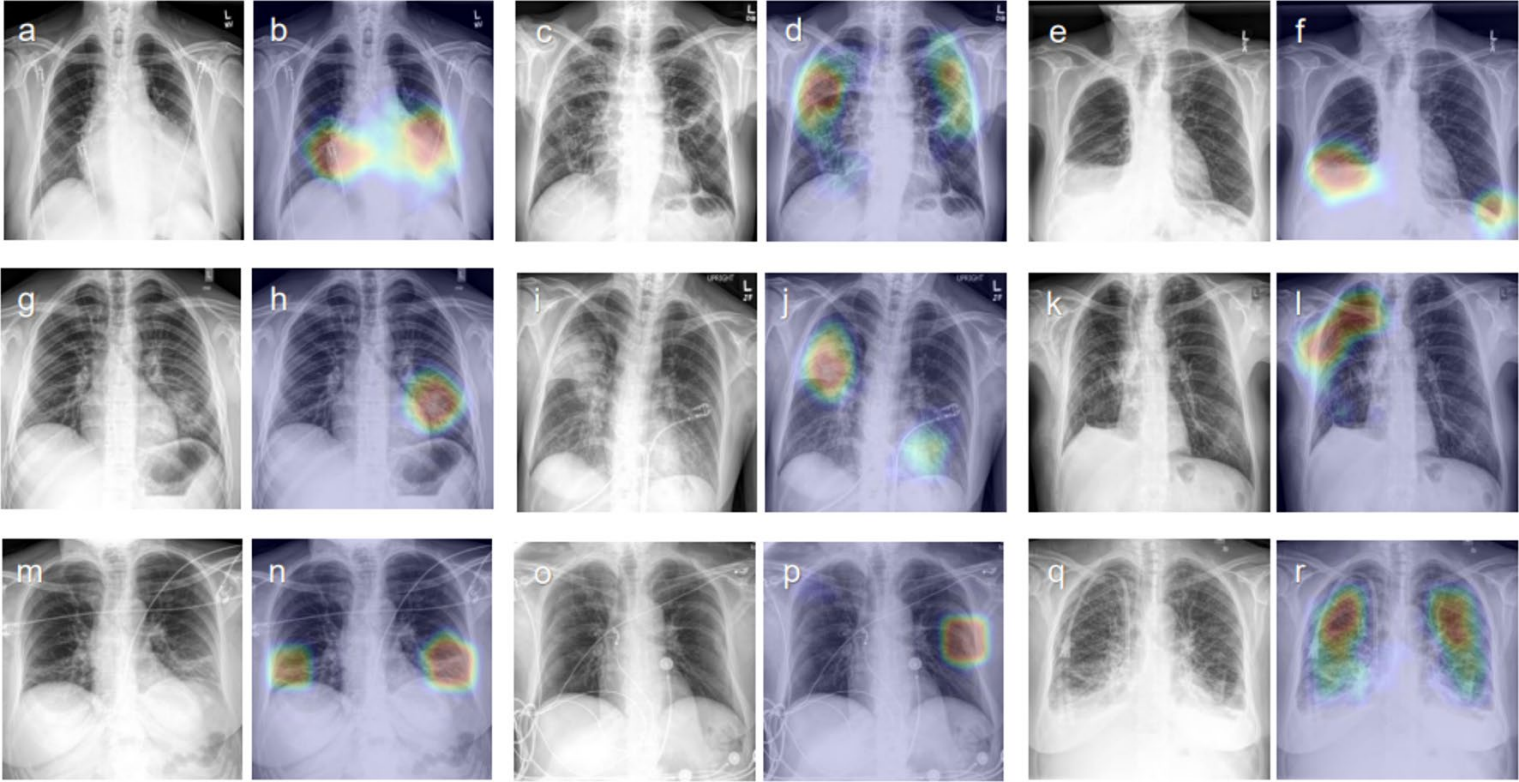
Representative class activation maps (CAMs) with correct lesion localization. (**a,b**) Cardiomegaly (PA), (**c,d**) Other interstitial opacity (PA), (**e,f**) Pleural effusion (PA), (**g,h**) Pneumonia (PA), (**i,j**) Pneumonia (AP), (**k,l**) Pneumothorax (PA), (**m,n**) Atelectasis (AP), (**o,p**) Fracture (AP), (**q,r**) Pulmonary edema (PA).

**Figure 2:**
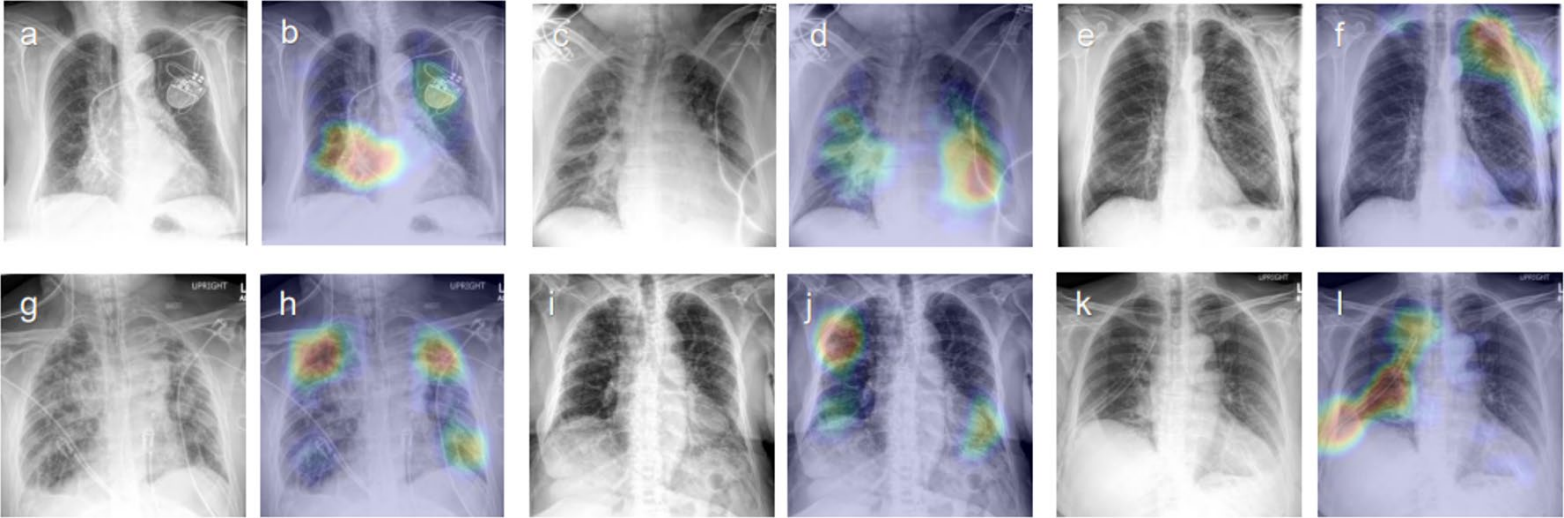
Representative class activation maps (CAMs) with incorrect lesion localization. (**a,b**) Cardiomegaly (PA), (**c,d**) Cardiomegaly (AP), (**e,f**) Pneumothorax (PA), (**g,h**) Other interstitial opacity (AP), (**i,j**) Other interstitial opacity (AP), (**k,l**) Pneumothorax (AP**)**. (**b**) Attention map captures the tip and body of the implantable cardioverter defibrillator (ICD) rather than the enlarged heart, (**d**) Attention map captures pulmonary edema along with the enlarged outline of heart in cardiomegaly, (**f**) Attention map captures subcutaneous emphysema along with pneumothorax, (**h,j**) Attention map failed to capture the full area of diffuse interstitial opacities, (**i**) Attention map captures chest tube instead of pneumothorax.

Spatial localization of abnormal radiographic features was performed using gradient-weighted class activation mapping (Grad-CAM)^7^. Figure 1 shows representative examples of attention maps that correctly localized the lesions. Figure 2 shows representative examples of attention maps that failed to correctly localize the lesions.

### Interpretability of xAI model predictions

**Figure 3:**
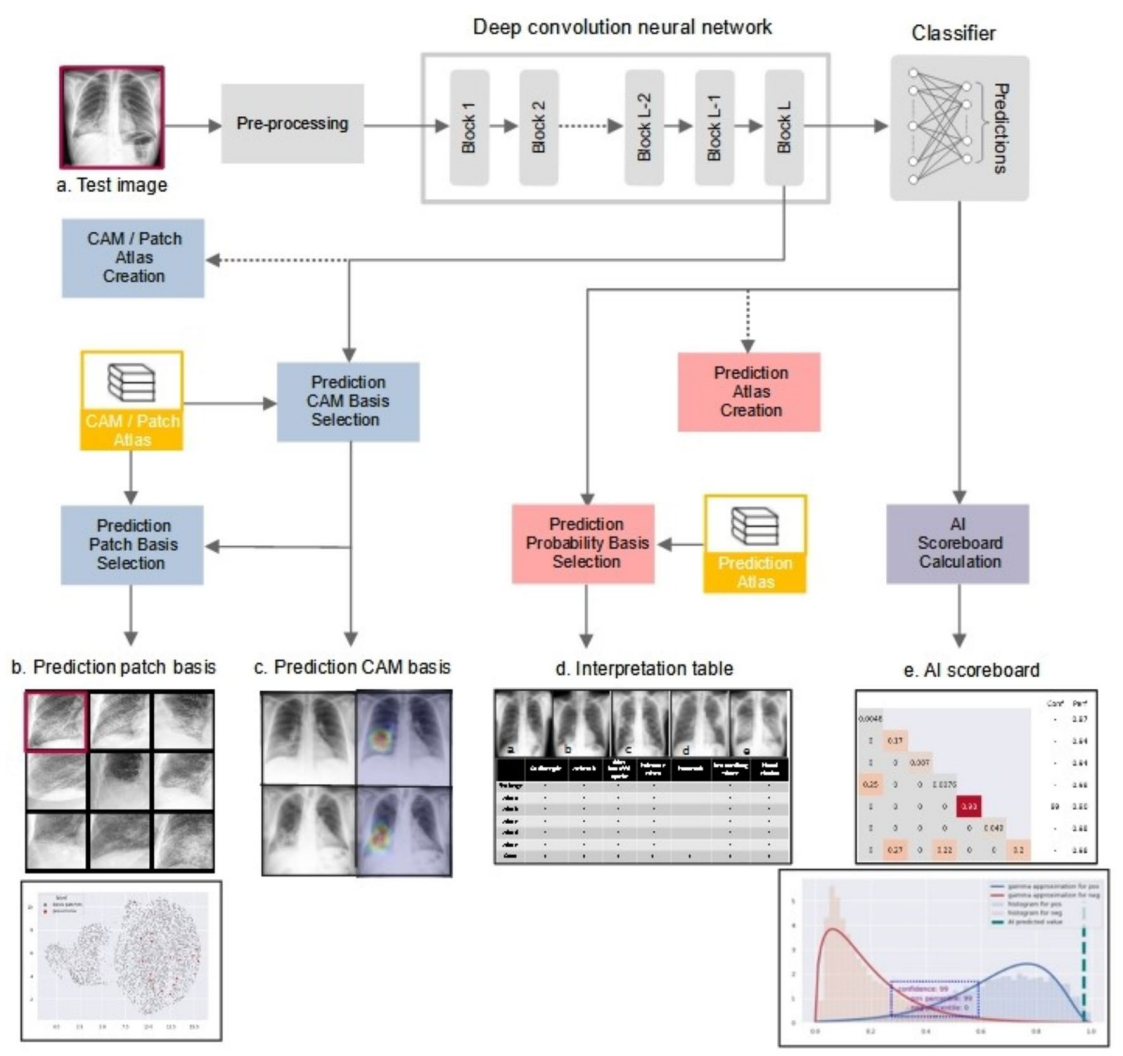
Schematic overview of CXR interpretation by our xAI model using a three-dimensional approach. The architecture of our CXR xAI model includes DenseNet-121 pre-trained DCNNs, (**a**) pipeline of pre-processing techniques, an Atlas creation module and prediction-based retrieval modules. The xAI model produces 3 types of outputs: (1) label prediction and attention map with corresponding feature patches selected from the Model-Derived Atlas (**b**,**c**). The first one on the upper left is a feature specific patch from the test CXR and the other eight are selected from the Atlas, which were closely located to the test patch on UMAP (**b**). The four CXR images were retrieved from the Atlas, which have similar overall characteristics with the test CXR (**c**). (2) an interpretation table displaying prediction probabilities for coexisting labels and comparable CXRs selected from the Model-Derived Atlas **(d)**, and (3) an AI scoreboard displaying prediction probabilities and calibrated classifier confidence, and histogram for AI prediction, positive and negative percentile (**e**).

We created a Model-Derived Atlas consisting of representative feature patches and CXR images from the training set^6^. For a test image, abnormal features are labelled by the classifier (Fig. 3a), Grad-CAM is applied to high confidence features identified by the AI scoreboard to generate an attention map for feature localization (Fig. 3c), and eight similar feature-specific patches are selected from the Model-Derived Atlas (Fig. 3b). Dimensionality reduction by uniform manifold approximation and projection (UMAP) shows that the test patch is close to corresponding patches from the Model-Derived Atlas in the two-dimensional embedding space.

**Figure 4:**
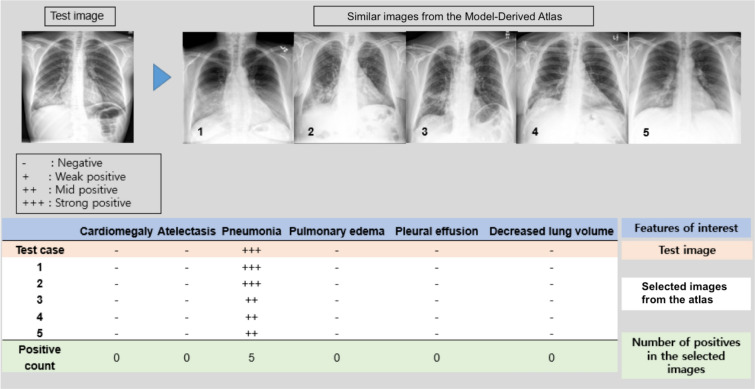
Interpretation table with similar CXRs selected from the Model-Derived Atlas. Visual comparison of the test image to similar CXRs with ground truth labels provides justification for model predictions. The table uses ‘−,’ ‘ + ,’ ‘ ++ ,’ and ‘ +++ ’ symbols to demonstrate similar combinations of pathological findings; prediction probability is ≥ 0.90 for ‘ +++ ’, < 0.90 and ≥ 0.80 for ‘ ++ ’, < 0.80 and ≥ 0.70 for ‘ + ’, and < 0.70 for ‘−’.

An interpretation table summarizes the prediction probabilities for coexistent and correlated features and displays comparable CXRs selected from the Model-Derived Atlas (Figs. 3d, 4). Presentation of similar CXRs with ground truth labels and corresponding prediction probabilities provides a reasonable basis for justification of the classification results. Clinicians may also select specific labels of interest for further characterization of a particular disease.

The AI scoreboard is a single diagram that displays calibrated classifier confidence for predicted features, overall model accuracy from the test set for predicted features, and correlation coefficients between selected features of interest (Fig. 3e, Supplementary Fig. 4). Highly reliable predictions can be identified from the AI scoreboard by high prediction probability and calibrated classifier confidence (≥ 0.9).

**Figure 5:**
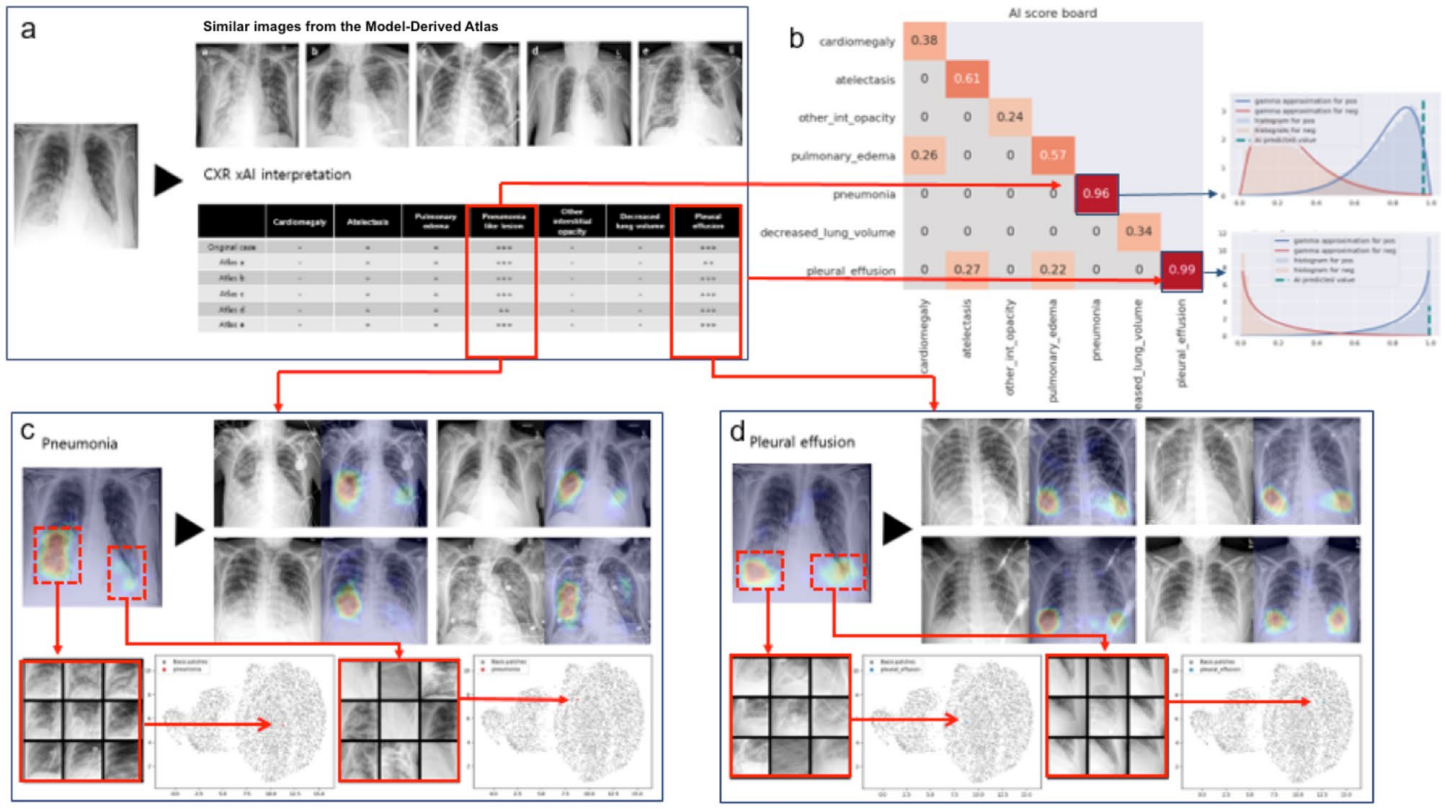
CXR interpretation by our xAI model for a patient who presented with respiratory infection. (**a**) The interpretation table shows 5 comparable CXRs selected from the Model-Derived Atlas and prediction probabilities for labels associated with respiratory infection. (**b**) Prediction probability was ≥ 0.90 on the AI scoreboard for pneumonia and pleural effusion. (**c,d**) Pneumonia and pleural effusion were correctly localized by Grad-CAM, and similar CXRs and patches were selected from the Model-Derived Atlas. UMAPs show that the test patches were close to corresponding patches from the Model-Derived Atlas, which supports that the testing patches can be classified as “pneumonia” and “pleural effusion”, respectively.

**Figure 6:**
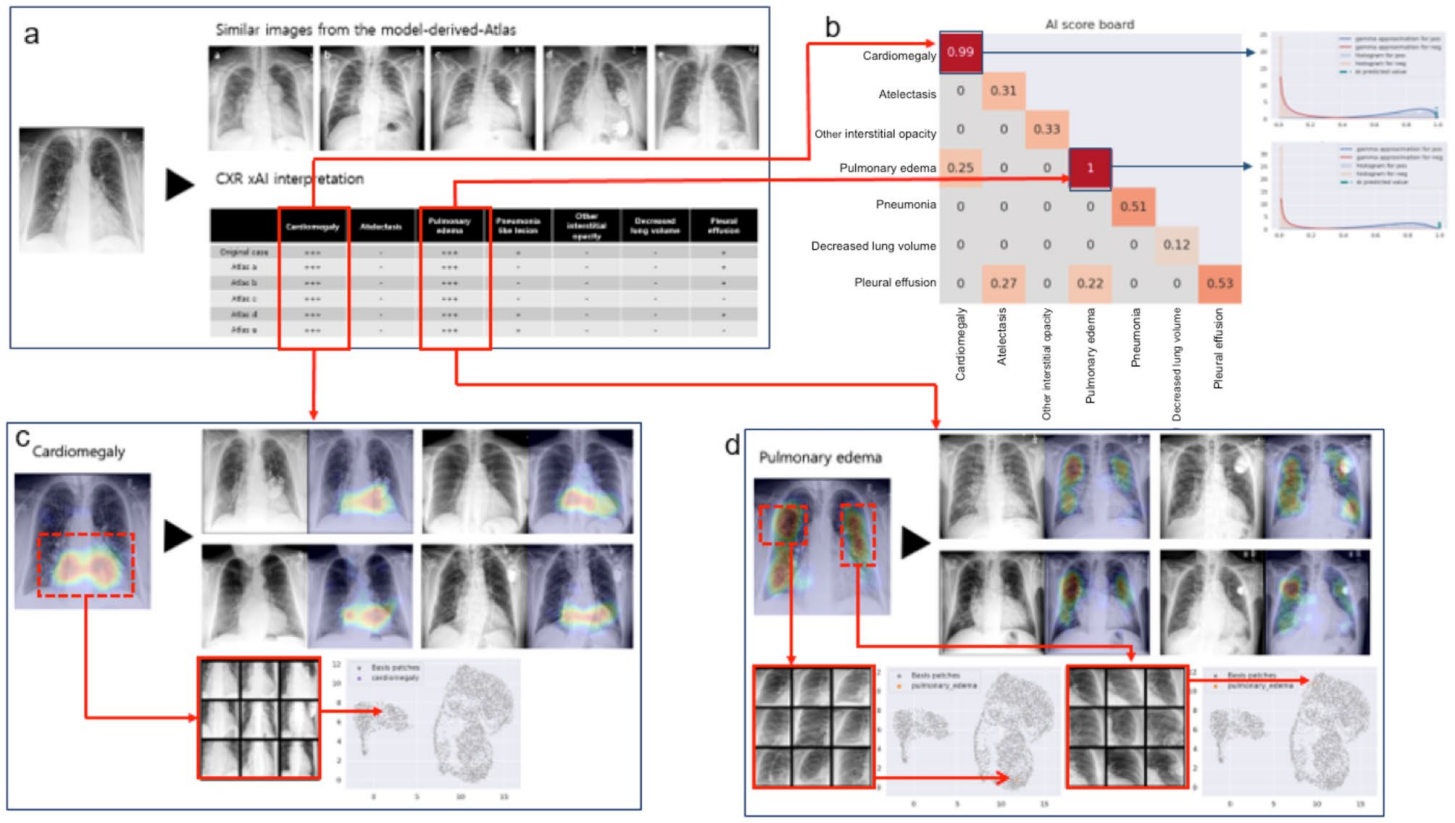
CXR interpretation by our xAI model for a patient who presented with heart failure. (**a**) The interpretation table shows 5 comparable CXRs selected from the Model-Derived Atlas and prediction probabilities for labels associated with heart failure. (**b**) Prediction probability was ≥ 0.90 on the AI scoreboard for cardiomegaly and pulmonary edema. (**c,d**) Cardiomegaly and pleural effusion were correctly localized by Grad-CAM, and similar feature CXRs and patches were selected from the Model-Derived Atlas. UMAPs show that the test patches were close to corresponding patches from the Model-Derived Atlas, which supports that the testing patches can be classified as “cardiomegaly” and “pulmonary edema”, respectively.

Figures 5 and 6 are illustrative examples demonstrating CXR interpretation by our xAI model for pneumonia and heart failure, respectively.

In Fig. 5a, the interpretation table displays high prediction probabilities for pneumonia and pleural effusion and low prediction probabilities for atelectasis and pulmonary edema, as well as 5 similar CXRs selected from the Model-Derived Atlas. Pneumonia and pleural effusion were identified as significant features, based on prediction probability and calibrated classifier confidence. Prediction probability for pneumonia and pleural effusion was 0.96 and 0.99, respectively, on the AI scoreboard (Fig. 5b). Pneumonia was correctly localized by Grad-CAM, and 4 similar pneumonia images were extracted from the Model-Derived Atlas (Fig. 5c). On UMAP, the test patch was closely located to eight corresponding feature-specific patches from the Model-Derived Atlas in the embedding space after dimensionality reduction (Fig. 5c). Pleural effusion was also correctly localized by Grad-CAM, and the test patch is likewise close to corresponding patches from the Model-Derived Atlas in the two-dimensional embedding space after dimensionality reduction (Fig. 5d).

In Fig. 6, the interpretation table displays high prediction probabilities for cardiomegaly and pulmonary edema and low prediction probabilities for pneumonia and pleural effusion. Cardiomegaly and pulmonary edema were identified as significant features, based on prediction probability and calibrated classifier confidence (Fig. 6a). Prediction probability for cardiomegaly and pulmonary edema was 0.99 and 1, respectively, on the AI scoreboard (Fig. 6b). The regions of interest were correctly localized by Grad-CAM and similar four images were extracted for each significant feature from the Model-Derived Atlas (Fig. 6c). Test patches were close to eight corresponding feature-specific patches from the Model-Derived Atlas in the two-dimensional embedding space after dimensionality reduction (Fig. 6c,d).

### Application of xAI model to COVID-19 for prediction of oxygen requirement

To evaluate the viability of a fine-tuning approach for efficient and scalable development of AI risk prediction models, we used our CXR xAI model to predict oxygen requirement in patients with COVID-19. A total of 1740 patients were diagnosed in the ED with COVID-19 by detection of SARS-CoV-2 in respiratory specimens by RT-PCR. There were 903 male and 837 female patients with a mean age of 59.3 ± 19.3 years (range: 16.0–107.0 years). Oxygen requirement was stratified using room air (RA), low flow oxygen (LFO,  ≤ 6L/min), high flow oxygen (HFO,  > 6L/min or non-invasive ventilation), and mechanical ventilation (MV) as 4 stages of severity.

A 2-step approach was used to predict oxygen requirement in COVID-19 patients at 24 and 72 h from the time of ED admission. In the first step, CXRs obtained at the time of ED admission were applied to the CXR xAI model to identify abnormal radiographic features. A random forest model was then fitted using 7 infection-associated radiographic labels (pneumonia, atelectasis, other interstitial opacity, pulmonary edema, pleural effusion, cardiomegaly, and decreased lung volume)^8–12^, reflecting the severity and pathophysiological characteristics of respiratory infection, and 8 clinical parameters (age, gender, heart rate, body temperature, systolic blood pressure, respiratory rate, peripheral oxygen saturation, and initial oxygen requirement).

Predictive performance improved for all stages of oxygen requirement when clinical information was included (Table 2). Supplementary Fig. 2 shows the ROCs for each prediction. Absence of pneumonia, absence of pulmonary edema, and absence of pleural effusion on CXR are predictive of low oxygen requirement at 24 and 72 h, whereas presence of pneumonia, presence of other interstitial opacity, and presence of pulmonary edema are predictive of high oxygen requirement at 24 and 72 h (Supplementary Fig. 3). Low initial oxygen requirement was most predictive of low oxygen requirement after 24 h, but radiographic findings were more important for predicting high oxygen requirement after 72 h (Supplementary Fig. 3).

**Table 2:** Performance metrics for prediction of oxygen requirement in patients with COVID-19 using the CXR xAI model with and without clinical information.

	Outcome	RF without clinical data						RF with clinical data					
		Cutoff value	Sensitivity	Specificity	PPV	NPV	Accuracy	Cutoff value	Sensitivity	Specificity	PPV	NPV	Accuracy
24 h	RA (N = 192)	0.504	0.729	0.786	0.791	0.723	0.756	0.326	0.938	0.767	0.811	0.916	0.852
	LFO (N = 141)	0.296	0.574	0.741	0.583	0.735	0.677	0.400	0.518	0.951	0.869	0.758	0.784
	HFO (N = 15)	0.100	0.133	0.986	0.236	0.964	0.951	0.032	0.533	0.974	0.471	0.980	0.956
	MV (N = 17)	0.014	0.588	0.902	0.227	0.978	0.888	0.030	0.765	0.908	0.289	0.988	0.901
72 h	RA (N = 166)	0.398	0.783	0.754	0.726	0.807	0.767	0.608	0.759	0.884	0.846	0.815	0.827
	LFO (N = 147)	0.386	0.320	0.858	0.603	0.652	0.641	0.322	0.497	0.872	0.723	0.720	0.721
	HFO (N = 20)	0.070	0.250	0.942	0.200	0.956	0.912	0.016	0.550	0.913	0.268	0.972	0.893
	MV (N = 32)	0.002	0.563	0.793	0.207	0.950	0.773	0.010	0.531	0.823	0.224	0.948	0.797

*RF* random forest, *RA* room air, *LFO* low flow oxygen, *HFO* high flow oxygen and *MV* mechanical ventilation.

In the second step, we derived the most likely stage of oxygen requirement at 24 and 72 h from the time of ED admission. For imbalanced classification, threshold tuning is a simple and straightforward approach for improving the performance of a classifier, and the geometric mean may be utilized to seek a balance between sensitivity and specificity^13^. Grid search was therefore performed on a held out test dataset to select threshold prediction probabilities for each stage of oxygen requirement to maximize the geometric mean of sensitivity and specificity. For each test case, we computed the difference between the output prediction probability and cut-off prediction probability values to find the stage with the largest positive difference.

Table 2 shows performance metrics for prediction of oxygen requirement at 24 and 72 h from the time of ED admission using radiographic risk predictors and radiographic plus clinical risk predictors. Conventional cardiorespiratory radiographic findings alone were predictive of RA at 24 h (0.729) and 72 h (0.783), LFO at 24 h (0.574) and MV at 24 h (0.588) and 72 h (0.563) from ED admission with reasonable sensitivity (Table 2). Addition of clinical risk predictors improved sensitivity for RA, HFO, and MV at 24 h from ED admission from 0.729, 0.133, and 0.588 to 0.938, 0.533, and 0.765, respectively, and also improved sensitivity for LFO and HFO at 72 h from ED admission from 0.320 and 0.250 to 0.497 and 0.550, respectively (Table 2).

**Figure 7:**
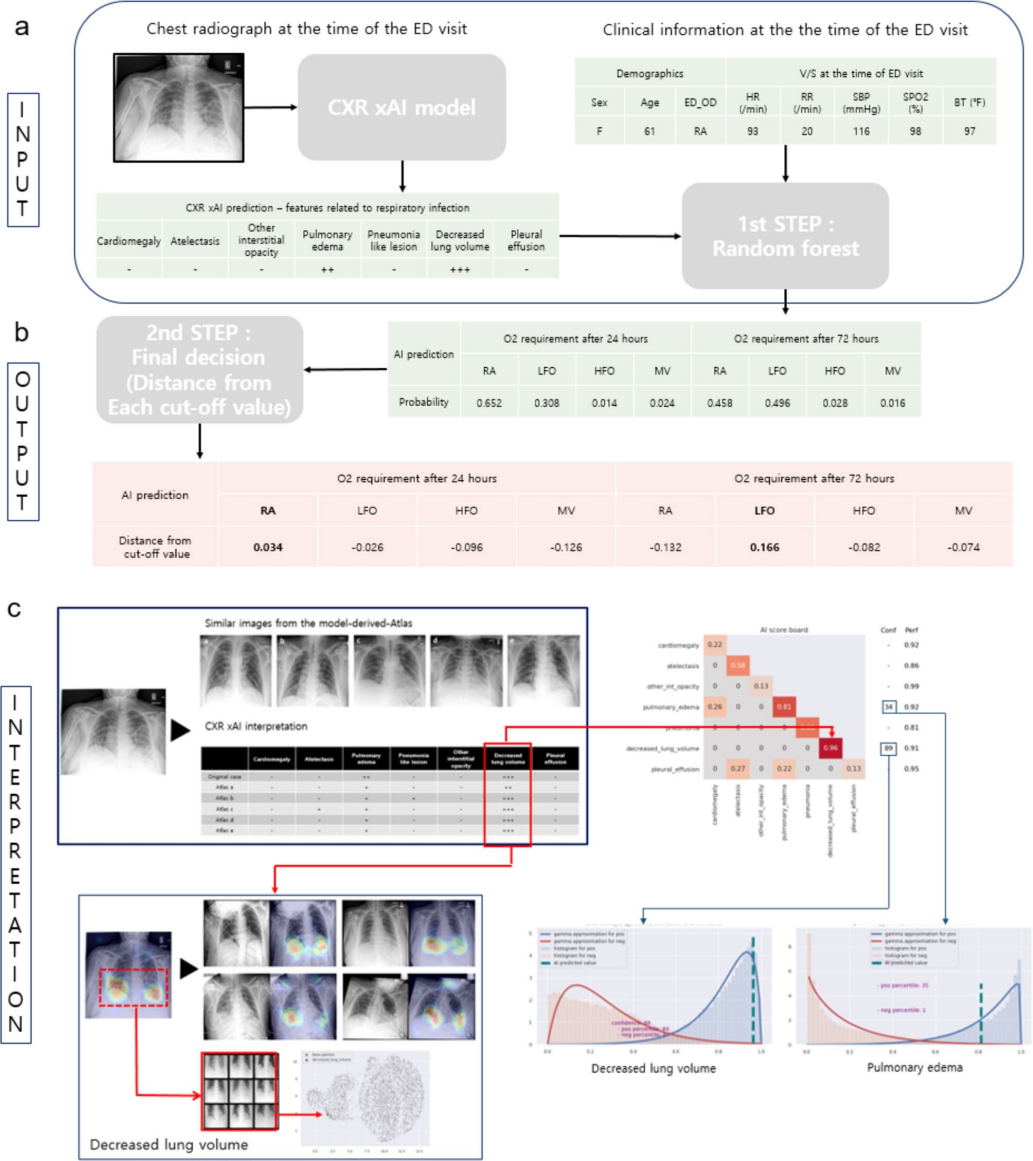
CXR interpretation and AI prediction of most likely stage of oxygen requirement at 24 and 72 h from the time of ED admission in patients with COVID-19. (**a**) Input: For a test case, prediction probabilities for each stage of oxygen requirement at 24 and 72 h from the time of ED admission are derived from the random forest model. (**b**) Output: The stage with the largest positive difference between prediction probability and the cut-off value is selected as the predicted stage of oxygen requirement. (**c**) Interpretation: Prediction probabilities for 7 infection-associated radiographic labels are summarized in the interpretation table and comparable CXRs were selected from the Model-Derived Atlas. Decreased lung volume was identified as a significant feature, based on high prediction probability and calibrated classifier confidence on the AI scoreboard. Feature localization with Grad-CAM and close location on UMAP to the similar feature patches from the Model-Derived Atlas provide visual evidence of decreased lung volume to support the prediction.

Figure 7 illustrates the process of oxygen requirement prediction for COVID-19 patients using clinical information and radiographic findings. A 61-year-old female patient was tolerant of RA at the time of admission to the ED, witConventional h stable vital signs and normal peripheral oxygen saturation. Our CXR xAI model did not identify infection-associated radiographic features except for pulmonary edema and decreased lung volume. Our COVID-19 xAI model predicted that the patient could be maintained on RA for 24 h, but LFO would be required after 72 h. Normal peripheral oxygen saturation and absence of pneumonia and pleural effusion on initial CXR were most predictive of stable respiratory status on RA at 24 h. However, decreased lung volume on initial CXR was a significant predictor of adjunctive oxygen therapy requirement after 72 h.

**Figure 8:**
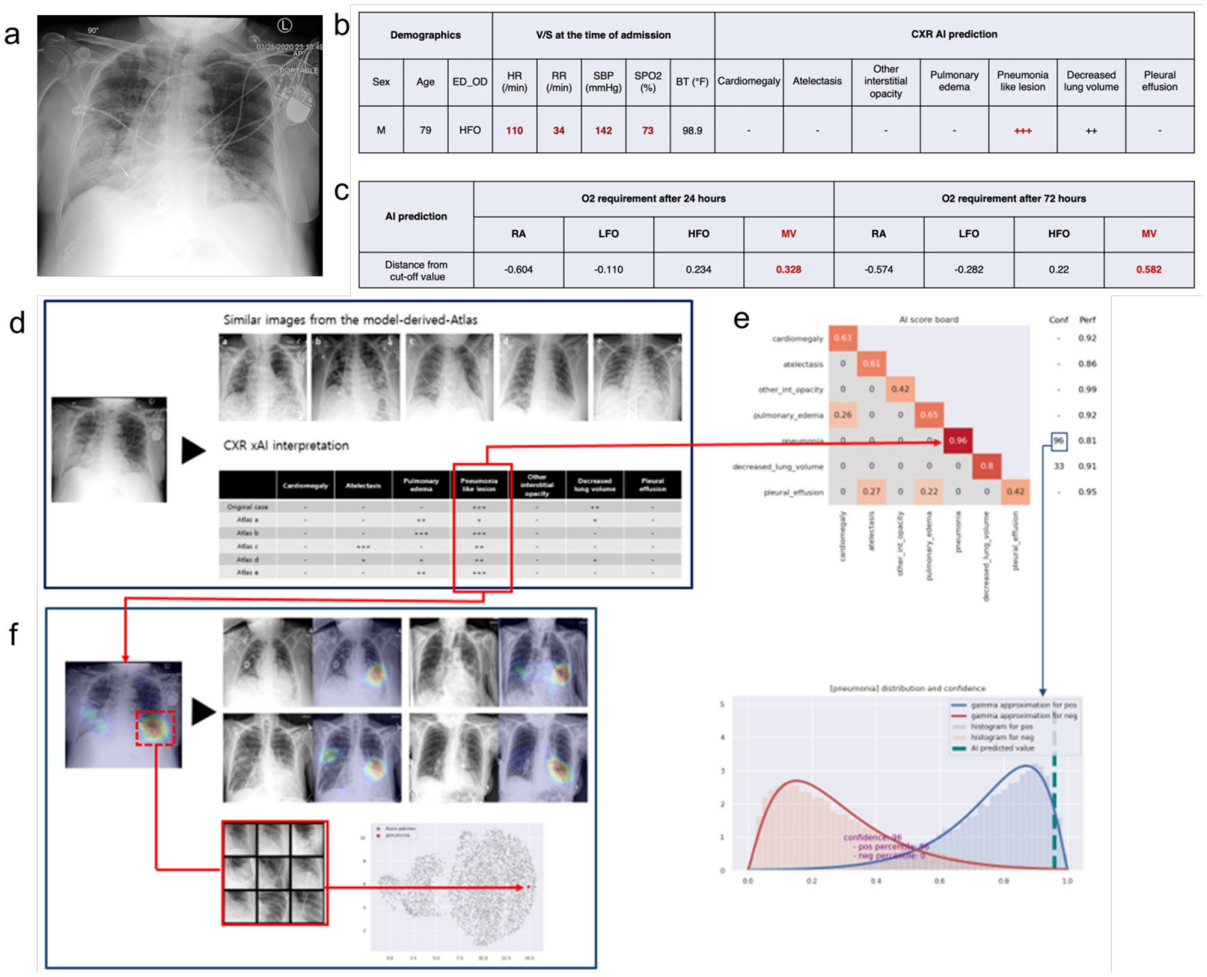
AP CXR of a 79-year-old man with COVID-19 and respiratory insufficiency and AI prediction of most likely stage of oxygen requirement at 24 and 72 h from the time of ED admission. (**a**) AP CXR obtained on ED admission, (**b**) Clinical information and infection-associated radiographic labels identified by our CXR xAI model, (**c**) Final prediction of oxygen requirement after 24 and 72 h. Our COVID-19 xAI model predicted that the patient would require MV after 24 and 72 h, (**d**) Prediction probabilities for 7 infection-associated radiographic labels are summarized in the interpretation table and 4 similar CXRs with characteristic findings for pneumonia were selected from the Model-Derived Atlas, (**e**) Pneumonia was identified as a significant feature, based on high prediction probability and calibrated classifier confidence on the AI scoreboard, (**f**) Pneumonia was correctly localized by Grad-CAM, and on UMAP, the test patch was closely located to corresponding patches from the Model-Derived Atlas in the embedding space after dimensionality reduction.

Figure 8 shows an example of severe COVID-19 infection requiring intensive respiratory support. A 79-year-old man with shortness of breath, elevated respiratory rate of 34 breaths/min, low peripheral oxygen saturation of 73%, and an elevated heart rate of 110 bpm required immediate initiation of HFO on admission to the ED. Our COVID-19 xAI model correctly predicted that MV would be required after 24 and 72 h, based on elevated respiratory rate, low peripheral oxygen saturation, and radiographic evidence of pneumonia on initial CXR.

## Discussion

To interpret CXRs, radiologists must first detect and characterize normal and abnormal lesions in order to assign the most appropriate diagnostic labels to these radiographic findings. Existing machine learning models have achieved comparable performance to practicing radiologists in detecting solitary and discrete radiographic findings, such as cardiomegaly, pleural effusion, and some focal lung opacities^14–21^. However, the radiographic appearance of other respiratory diseases is quite variable. Different diseases may have similar radiographic appearances, and the characteristic findings of a specific disease may only emerge later in the disease course. As such, classification performance is poorer for most pulmonary opacities, including pneumonia, pulmonary edema, atelectasis, and interstitial lung disease^14–21^. This reflects inherent limitations of the CXR itself as a diagnostic tool and associated interobserver variability.

Variability and overlap in radiographic appearance gives rise to unavoidable prediction uncertainty. The incidence of some respiratory diseases may also be low, making it difficult to obtain sufficient data for model training. For this reason, calibrated classifier confidence should be reported to qualify a prediction, and explainability techniques such as feature localization should be employed. Atlas-based explanations using nearest-neighbour image retrieval methods are practicable and frequently preferred by expert radiologists^22–24^.

Integration of coexistent radiographic findings is required for formulation of a disease diagnosis and assessment of disease severity. Our CXR xAI model retrieves and displays comparable CXRs from the Model-Derived Atlas with ground truth labels and corresponding prediction probabilities, permitting visual and probabilistic comparison to justify model predictions. This method has potential to build trust with physicians and improve clinical decision-making^25,26^. Labels of interest may be selected by physicians and incorporated into the interpretation table. In this work, pneumonia, atelectasis, other interstitial opacity, pulmonary edema, pleural effusion, decreased lung volume, and cardiomegaly were selected as labels of interest for predicting the severity of COVID-19 infection in ED patients.

Fully automated risk prediction using radiographic features has not been well-investigated. Only a few machine learning models have been recently developed to predict mortality or critical illness in patients with COVID-19^1,2,4,5^. Risk prediction using “deep learning extracted” radiographic features sidesteps the need for accurate image annotation. However, these black box models suffer greatly from lack of accountability and interpretability, which are crucial for responsible implementation of AI systems in safety–critical applications^2,3,5^. According to the authors, regions of airspace and interstitial opacification were apparently highlighted by saliency mapping. Such generalized observations may engender a false sense of safety and perpetuate confirmation bias^27^.

Our risk prediction model considers and validates the relevance of cardiorespiratory comorbidities, such as decreased lung volume, pleural effusion, pulmonary edema, atelectasis, and cardiomegaly, to COVID-19 disease severity. For example, as illustrated by our case vignette, decreased lung volume is relevant for prediction of impending oxygen requirement (Fig. 7). In addition, radiographic findings were more important than clinical information for predicting high oxygen requirement at 72 h (Supplementary Fig. 3). In comparison, previously developed full training risk prediction models have focused only on a few visual aspects of pulmonary opacities, such as “extent of lung involvement” and “degree of opacification” as candidate radiographic predictors^1,4^ and do not capture a broad scope of cardiorespiratory pathophysiological manifestations and comorbidities. Moreover, “extent of lung opacification” may not be superior or even non-inferior to well-established clinical predictors, such as peripheral oxygen saturation and C-reactive protein in serum^1^.

At the derived best operating points, our full RF model demonstrates high sensitivity for RA and MV at 24 h from ED admission, and high sensitivity for RA at 72 h from ED admission, with corresponding high specificity, and may potentially be deployed in the ED to guide patient triage into low risk and high risk categories (Table 2). Unsurprisingly, predictive performance was relatively poorer at 72 h from ED admission. Time-dependent predictors such as changes in vital signs and evolution of radiographic findings may be more predictive of delayed respiratory deterioration. Lower sensitivity for LFO at both interrogated time points may reflect more variable indications for LFO administration, as well as wider availability and lower cost, compared to HFO and MV.

While sensitivity and specificity are characteristics of a test*,* PPV and NPV are influenced by prevalence of the disease or outcome in the test population. As such, PPV for HFO and MV in our cohort is low. In order to provide clinical users with reliable estimates PPV and NPV, which may vary significantly from smaller community hospitals to larger tertiary referral centers, a pre-deployment phase, in which a risk prediction model runs in the background to generate population specific performance metrics, appears to be necessary.

Future studies should validate the performance of our CXR xAI model on other clinically important risk prediction tasks. For example, fully automated prediction of unplanned return to the ED with or without hospitalization or intensive care unit (ICU) admission in patients with acute decompensated heart failure, patients with acute exacerbation of chronic obstructive pulmonary disease (COPD), and elderly patients with community acquired pneumonia may be investigated, as these indicators are key measures of the quality and safety of care provided by an ED^28^.

### Limitations

This study has several limitations. Training data was collected from a single institution. This affects the generalizability of our CXR xAI model and COVID-19 xAI model to patient cohorts from other institutions due to population differences and use of different imaging equipment. Model performance was also not compared to the performance of practicing radiologists.

Approximately 270,000 CXRs were used in model development. However, we were unable to collect sufficient training data for certain features, such as emphysema and cavity/cyst. Nevertheless, our CXR xAI model performed well even in these categories. Training data may be supplemented through continuous updates in the future.

Ground truth labels were assigned for classification of 20 abnormal radiographic features based on keywords extracted from the radiology reports. Common limitations arising from use of natural language processing (NLP) for ground truth labelling include usage of diverse descriptions for similar lesions, usage of implicative terms, and use of non-specific language by radiologists. It is necessary for clinicians to develop standardized radiology reports for more generalizable and unbiased AI model development.

We used a cohort of COVID-19 patients to provide an example of how our CXR xAI model can be applied in a real world clinical setting. However, our study only includes patients who visited the ED in the first 2 months of the COVID-19 outbreak, and additional clinical data, such as comorbidities and follow-up clinical outcomes, was not available.

## Conclusion

We have developed an auditable CXR xAI model, which captures a broad scope of cardiorespiratory pathophysiological manifestations. and comorbidities. Our CXR xAI model may potentially be broadly and efficiently applied via a fine-tuning approach in real world clinical situations to produce fully automated risk and outcome predictions.

## Methods

### Data collection

CXRs obtained at our institution from February 2015 to February 2019 were identified from our Radiology Information System (RIS) and Picture Archiving and Communication System (PACS). A total of 440,852 studies from 117,195 patients were retrospectively retrieved after excluding studies without an associated radiology report and studies without view position information. After automated NLP data mining and clean-up steps, we acquired 151,700 AP views from 49,096 patients and 90,023 PA views from 69,404 patients.

### Label extraction from radiology reports

We developed an automated NLP program for label extraction from radiology reports using binary classification and a rule-based approach. Data processing was performed in 3 stages. First, we constructed a dictionary for extraction of radiological terms using RadLex Lexicon ontology data (version 4.1)^29^ and manually added terms based on manual review of the radiology reports. Second, we extracted lemmatized terms from the dictionary, checked for negation and double negation, and modified the result to prevent false positives and false negatives. Third, we constructed a “clean negative” dataset containing categorically negative labels, such as “no pulmonary edema or pneumonia”. This dataset was used to check for conflicting descriptions within a radiology report due to physician error and for labelling errors from the first and second steps. Negative labels from the second step but not in the “clean negative” dataset were ignored in the NLP extraction performance test and CNN training.

For these steps, we used NLTK, tokenizer for sentence parsing, Spacy for word tokenizing, and Parts of Speech (POS) for tagging and dependency parsing. Using our rule-based NLP program, 4,383 feature-related keywords were automatically extracted from radiology reports. Feature-related keywords were then categorized using 20 feature labels, and assigned a “positive”, “negative” or “ignore” value. Supplementary Table 1 shows the prevalence of each feature label in the train, validation, and test sets and Supplementary Table 2 shows high accuracy and precision for extraction of the 20 feature labels using our NLP program.

### Multilabel classification

We built a keyword library with RadLex Lexicon ontology data and terms extracted from the radiology reports. Each keyword was reviewed and categorized with consensus by 3 Korean board-certified radiologists and 3 physicians. Finally, the features were classified into 7 anatomy-based categories (Lung volume, Lung opacity, Hilum/mediastinum, Pleural lesion, Bone, Diaphragm, and Foreign body). Lung volume was further classified into 2 subcategories (Increased lung volume and Decreased lung volume), lung opacity was further classified into 7 subcategories (Atelectasis, Pneumonia-like lesion hereinafter referred to as Pneumonia, Nodule/Mass, Pulmonary edema, and Other interstitial opacity); Hilum/mediastinum was further classified into 4 subcategories (Hilar area, Mediastinum, Cardiomegaly, and Aorta); Pleural lesion was further classified into 3 subcategories (Pleural effusion, Pneumothorax, and Other pleural lesion); and Bone was further classified into 2 subcategories (Fracture and Non-fracture). Each CXR may contain multiple labels.

### Comprehensive feature set for a specific disease by regrouping of 20 labels

Our xAI model produces predictions for 20 labels. In comparison, a maximum of 14 labels have been used in prior studies^14–21^. To use these labels for description of specific diseases, we identified groups of abnormal features associated with specific conditions or diseases, which can be displayed in the interpretation table. For example, labels associated with respiratory infection may include lung opacity-related features such as pneumonia and atelectasis, as well as additional features as complications, such as pleural effusion and pulmonary edema. To evaluate for acute exacerbation of heart failure, cardiomegaly, pulmonary edema, and pleural effusion should be displayed. If traumatic chest injury is suspected, predictions for bone fracture, pneumothorax, and pleural effusion are relevant, and additional predictions for diaphragm, mediastinum, and cardiomegaly may also be necessary, depending on the clinical context.

Regrouping of abnormal features may be performed as required by physicians in different clinical settings, and more comprehensive xAI models can also be developed by addition of clinical information, such as age, gender, respiratory symptoms, vital signs, and laboratory results. In this study, we developed a comprehensive xAI model for prediction of oxygen requirement in COVID-19 patients using 7 radiographic features and 8 clinical parameters as predictors.

### Test set annotation

We randomly selected 1000 PA views and 1000 AP views for model testing. CXRs in the test set were annotated by 3 United States board-certified radiologists at our institution, with 1–6 years of experience in emergency radiology. For each label, majority voting was employed to resolve annotator disagreement and derive a single ground truth. To annotate cases in the test set, we used MarkIt, a web-based annotation tool (Supplementary Fig. 5), as described in our previous study^6,30^.

### Model development

#### Explainable AI algorithm (xAI)

A detailed description of xAI model development, including CNN training, ensemble model construction, and distribution Atlas creation, is provided in our previous study^6^.

#### Patch Atlas creation based on CAM ensemble method

We developed a CAM ensemble method for patch atlas creation to improve the localization performance of CAM. For each label, representative features from the training set were included in the patch atlas if the corresponding prediction probability was ≥ 0.9. Feature patches were applied to a cosine metric-based UMAP model and coordinates for each feature patch in the two-dimensional embedding space were obtained^6,31^.

#### Interpretation table

Multilabel classification output for the test image is summarized in the interpretation table using symbols to demonstrate similar combinations of pathological findings, where prediction probability is ≥ 0.90 for ‘ +  +  + ’, < 0.90 and ≥ 0.80 for ‘ +  + ’, < 0.80 and ≥ 0.70 for ‘ + ’, and < 0.70 for ‘-’. N-nearest images are selected from the Model-Derived Atlas based on Euclidean distance from an L-dimensional probability vector of the test image, as shown in Fig. 4, where N = 5.

1 $$ \Psi_{pb} = \, \{ \Omega_{pb} \left( {1} \right), \ldots ,\Omega_{pb} \left( N \right)\} , $$

where ${\Omega }_{pb}=Sort\left(||\left(\overline{p }-{A}_{P}\left(i\right)\right)\cdot \overline{c }|{|}_{2}\right) for i=1, \dots , n({A}_{P})$, $\overline{\mathrm{c} }$ is an L-dimensional vector with one for the target classes and zeros for the others, $\overline{p }$ is an L-dimensional probability vector of the test image, and ${A}_{P}(n)$ denotes $n$-th probability vector in our probability atlas. $\Omega$ denotes the set with elements of a sorted index, $\Omega \left(m\right)$ is $m$ th index, and $\rho$ means the selected group.

#### AI scoreboard

The AI scoreboard displays essential execution metrics including prediction probabilities, calibrated classifier confidence, and correlation between classes, as shown in Supplementary Fig. 6.

Prediction probabilities and correlation between classes were computed in a single inference step, in which Pearson correlation coefficients were used to calculate the correlation between class weights from the trained neural network.

Calibrated classifier confidence represents how much we can trust the prediction given the prediction probability. It is calculated as the difference between a percentile from the probability density distribution for the positive class and a percentile from probability density distribution for the negative class.

2 $${Confidence}_{P} =\mathrm{max}\left({f}_{P}^{c}({y}^{C})-{(1 - f}_{N}^{c}({y}^{C})), 0\right),$$

3 $${Confidence}_{N}=\mathrm{max}\left({(1-f}_{N}^{c}({y}^{C})-{f}_{P}^{c}({y}^{C}), 0\right),$$

where ${y}^{C}$ is the predicted probability for the c-class, ${f}_{P}^{c}({y}^{C})$ is a percentile from the probability density distribution for the positive class, and ${f}_{N}^{c}({y}^{C})$ is a percentile from the probability density distribution for the negative class.

#### Application to COVID-19

We identified patients who visited the ED between March 2, 2020 and May 7, 2020 and were diagnosed with the SARS-CoV-2 virus by positive RT-PCR assay. Among the 1798 patients, 58 patients were excluded because an admission CXR was not available. We randomly selected 1375 patients (80%) for model development and 365 patients (20%) for model testing. Demographic and clinical data were obtained including age, gender, systolic blood pressure, heart rate, respiratory rate, body temperature, peripheral oxygen saturation, initial oxygen requirement, and oxygen requirement at 24 and 72 h from the time of admission to the ED. Oxygen requirement was recorded as room air (RA), low flow oxygen (LFO, ≤ 6L/min), high flow oxygen (HFO, > 6L/min or non-invasive ventilation), or mechanical ventilation (MV). Supplementary Table 3 summarizes the demographic and clinical characteristics of COVID-19 patients in our study.

Using predictions from our pre-trained CXR xAI model for 7 radiographic labels (Cardiomegaly, Atelectasis, Pulmonary edema, Pneumonia, Other interstitial opacity, Decreased lung volume, and Pleural effusion) and 8 clinical parameters (age, gender, heart rate, body temperature, systolic blood pressure, respiratory rate, peripheral oxygen saturation, and initial oxygen requirement) as inputs, we trained a random forest model using 500 trees in the forest, Gini criteria to measure the quality of a split, and bootstrap samples when building trees.

#### IRB approval

IRB approval for retrospective analysis was obtained from our institution (IRB approval #2019P002432). Informed consent was waived due to the retrospective nature of the study.

#### Statistical analysis and evaluation

Model performance was evaluated using AUROC, sensitivity (same as recall), specificity, positive predictive value (PPV, same as precision), negative predictive value (NPV), and F1 score, with 95% confidence intervals (CIs) for these 7 performance metrics.

## Supplementary Information

Supplementary Information.

## Data Availability

Data sharing is restricted by our institution’s policies. De-identified patient level data for participants in this study, our statistical analysis plan, and the statistical coding can be made available with approval of the IRB. Requests should be made to the corresponding author.
