# Supplementary material for: Prediction of oxygen requirement in patients with COVID-19 using a pre-trained chest radiograph xAI model: efficient development of auditable risk prediction models via a fine-tuning approach
Source: Sci Rep. 2022 Dec 7;12:21164. doi: 10.1038/s41598-022-24721-5 (PMC9729627; doi:10.1038/s41598-022-24721-5)
Supplement: Supplementary file 1 — Supplementary Information. [file 41598_2022_24721_MOESM1_ESM.pdf]

AP view	Train set						Validation set						Test set			
	Positive		Uncertain		Negative		Positive		Uncertain		Negative		Positive		Negative	
Fracture	3,446	4.8%	521	0.7%	68,171	94.5%	576	4.1%	89	0.6%	13,364	95.3%	12	1.2%	988	98.8%
Non-fracture	1,275	1.8%	26	0.0%	70,837	98.2%	177	1.3%	6	0.0%	13,846	98.7%	5	0.5%	995	99.5%
Diaphragm	3,293	4.6%	116	0.2%	68,729	95.3%	580	4.1%	30	0.2%	13,419	95.7%	8	0.8%	992	99.2%
Foreign body	44,034	61.0%	2,487	3.4%	25,617	35.5%	9,866	70.3%	440	3.1%	3,723	26.5%	677	67.7%	323	32.3%
Aorta	4,638	6.4%	351	0.5%	67,149	93.1%	716	5.1%	51	0.4%	13,262	94.5%	63	6.3%	937	93.7%
Cardiomegaly	10,583	14.7%	1,904	2.6%	59,651	82.7%	2,171	15.5%	407	2.9%	11,451	81.6%	227	22.7%	773	77.3%
Hilar area	266	0.4%	205	0.3%	71,667	99.3%	39	0.3%	29	0.2%	13,961	99.5%	2	0.2%	998	99.8%
Mediastinum	1,423	2.0%	449	0.6%	70,266	97.4%	255	1.8%	88	0.6%	13,686	97.6%	16	1.6%	984	98.4%
Cavity/Cyst	325	0.5%	110	0.2%	71,703	99.4%	116	0.8%	18	0.1%	13,895	99.0%	6	0.6%	994	99.4%
Emphysema	982	1.4%	2,261	3.1%	68,895	95.5%	170	1.2%	480	3.4%	13,379	95.4%	2	0.2%	998	99.8%
Atelectasis	36,785	51.0%	407	0.6%	34,946	48.4%	7,734	55.1%	126	0.9%	6,169	44.0%	554	55.4%	446	44.6%
Nodule/mass	3,663	5.1%	388	0.5%	68,087	94.4%	575	4.1%	49	0.3%	13,405	95.6%	18	1.8%	982	98.2%
Other interstitial opacity	2,334	3.2%	102	0.1%	69,702	96.6%	452	3.2%	20	0.1%	13,557	96.6%	8	0.8%	992	99.2%
Pulmonary edema	18,620	25.8%	2,921	4.0%	50,597	70.1%	4,568	32.6%	504	3.6%	8,957	63.8%	276	27.6%	724	72.4%
Pneumonia	19,581	27.1%	3,209	4.4%	49,348	68.4%	4,322	30.8%	579	4.1%	9,128	65.1%	174	17.4%	826	82.6%
Decreased lung volume	17,539	24.3%	879	1.2%	53,720	74.5%	3,352	23.9%	187	1.3%	10,490	74.8%	174	17.4%	826	82.6%
Increased lung volume	820	1.1%	16	0.0%	71,302	98.8%	158	1.1%	4	0.0%	13,867	98.8%	0	0.0%	1,000	100.0%
Other pleural lesions	1,678	2.3%	1,942	2.7%	68,518	95.0%	329	2.3%	348	2.5%	13,352	95.2%	4	0.4%	996	99.6%
Pleural effusion	23,020	31.9%	263	0.4%	48,855	67.7%	5,759	41.1%	51	0.4%	8,219	58.6%	348	34.8%	652	65.2%
Pneumothorax	5,485	7.6%	1,612	2.2%	65,041	90.2%	1,361	9.7%	351	2.5%	12,317	87.8%	71	7.1%	929	92.9%

PA view	Train set						Validation set						Test set			
	Positive		Uncertain		Negative		Positive		Uncertain		Negative		Positive		Negative	
Fracture	1,919	2.9%	474	0.7%	64,155	96.4%	372	3.0%	97	0.8%	11,785	96.2%	31	3.1%	969	96.9%
Non-fracture	2,787	4.2%	25	0.0%	63,736	95.8%	524	4.3%	7	0.1%	11,723	95.7%	10	1.0%	990	99.0%
Diaphragm	2,171	3.3%	132	0.2%	64,245	96.5%	393	3.2%	19	0.2%	11,842	96.6%	15	1.5%	985	98.5%
Foreign body	16,011	24.1%	1,189	1.8%	49,348	74.2%	3,208	26.2%	191	1.6%	8,855	72.3%	244	24.4%	756	75.6%
Aorta	3,272	4.9%	403	0.6%	62,873	94.5%	609	5.0%	85	0.7%	11,560	94.3%	167	16.7%	833	83.3%
Cardiomegaly	4,289	6.4%	1,001	1.5%	61,258	92.1%	805	6.6%	170	1.4%	11,279	92.0%	104	10.4%	896	89.6%
Hilar area	197	0.3%	97	0.1%	66,254	99.6%	37	0.3%	18	0.1%	12,199	99.6%	11	1.1%	989	98.9%
Mediastinum	794	1.2%	388	0.6%	65,366	98.2%	121	1.0%	87	0.7%	12,046	98.3%	8	0.8%	992	99.2%
Cavity/Cyst	221	0.3%	78	0.1%	66,249	99.6%	38	0.3%	19	0.2%	12,197	99.5%	3	0.3%	997	99.7%
Emphysema	830	1.2%	337	0.5%	65,381	98.2%	159	1.3%	61	0.5%	12,034	98.2%	2	0.2%	998	99.8%
Atelectasis	12,905	19.4%	245	0.4%	53,398	80.2%	2,539	20.7%	40	0.3%	9,675	79.0%	235	23.5%	765	76.5%
Nodule/mass	3,981	6.0%	503	0.8%	62,064	93.3%	733	6.0%	92	0.8%	11,429	93.3%	36	3.6%	964	96.4%
Other interstitial opacity	4,047	6.1%	209	0.3%	62,292	93.6%	780	6.4%	37	0.3%	11,437	93.3%	12	1.2%	988	98.8%
Pulmonary edema	2,417	3.6%	4,121	6.2%	60,010	90.2%	492	4.0%	783	6.4%	10,979	89.6%	35	3.5%	965	96.5%
Pneumonia	8,602	12.9%	4,836	7.3%	53,110	79.8%	1,663	13.6%	939	7.7%	9,652	78.8%	123	12.3%	877	87.7%
Decreased lung volume	4,530	6.8%	274	0.4%	61,744	92.8%	833	6.8%	50	0.4%	11,371	92.8%	23	2.3%	977	97.7%
Increased lung volume	2,290	3.4%	13	0.0%	64,245	96.5%	422	3.4%	4	0.0%	11,828	96.5%	8	0.8%	992	99.2%
Other pleural lesions	1,659	2.5%	1,372	2.1%	63,517	95.4%	370	3.0%	261	2.1%	11,623	94.9%	8	0.8%	992	99.2%
Pleural effusion	7,044	10.6%	299	0.4%	59,205	89.0%	1,512	12.3%	57	0.5%	10,685	87.2%	136	13.6%	864	86.4%
Pneumothorax	885	1.3%	388	0.6%	65,275	98.1%	155	1.3%	68	0.6%	12,031	98.2%	9	0.9%	991	99.1%

Supplementary Table 1. Summary statistics for train, validation, and test sets. Number of images with percentages are reported, unless otherwise specified. *Reproduced from Kim (2022)*[6].

Label	Accuracy (%)	Precision (%)	Recall (%)	F1 (%)
Fracture	100.0	100.0	100.0	100.0
Non-fracture	100.0	100.0	100.0	100.0
Diaphragm	100.0	100.0	100.0	100.0
Foreign body	100.0	100.0	100.0	100.0
Aorta	100.0	100.0	100.0	100.0
Cardiomegaly	100.0	100.0	100.0	100.0
Hilar area	100.0	100.0	100.0	100.0
Mediastinum	100.0	N/A	N/A	N/A
Cavity/Cyst	100.0	N/A	N/A	N/A
Emphysema	100.0	N/A	N/A	N/A
Atelectasis	100.0	100.0	100.0	100.0
Nodule/mass	99.1	50.0	100.0	66.7
Other interstitial opacity	100.0	100.0	100.0	100.0
Pulmonary edema	99.1	94.7	100.0	97.3
Pneumonia	100.0	100.0	100.0	100.0
Decreased lung volume	100.0	100.0	100.0	100.0
Increased lung volume	100.0	100.0	100.0	100.0
Other pleural lesions	100.0	100.0	100.0	100.0
Pleural effusion	100.0	100.0	100.0	100.0
Pneumothorax	100.0	100.0	100.0	100.0

Supplementary Table 2. Performance of the automated labeller on the report evaluation set for mention extraction, negation detection, and uncertainty detection tasks.

COVID19 dataset		P-value (MGH training vs test)	MGH (training set) N=1,375	MGH (test set) N=365
Sex	M	0.086	699 (50.8%)	204 (55.9%)
	F		676 (49.2%)	161 (44.1%)
Age	Mean, y	0.145	58.956±19.399	60.614 ± 19.094
Initial O_2	RA	0.805	894 (65.0%)	243 (66.6%)
	LFO		397 (28.9%)	99 (27.1%)
	HFO		84 (6.1%)	23 (6.3%)
	MV		0 (0%)	0 (0.0%)
Outcome	Admission	0.511	955 (69.5%)	260 (71.2%)
	Mortality	0.421	140 (10.2%)	32 (8.8%)
O_2 24hr	RA	0.229	745 (54.2%)	192 (52.6%)
	LFO		469 (34.1%)	141 (38.6%)
	HFO		67 (4.9%)	15 (4.1%)
	MV		94 (6.8%)	17 (4.7%)
O_2 72hr	RA	0.095	660 (48.0%)	166 (45.5%)
	LFO		468 (34.0%)	147 (40.3%)
	HFO		109 (7.9%)	20 (5.5%)
	MV		138 (10.0%)	32 (8.8%)

Supplementary Table 3. Demographic and clinical characteristics of patients with COVID-19. Number of patients with percentages in parentheses and P-values from comparison of the training set and test set are reported. *RA=room air, LFO=low flow oxygen, HFO=high flow oxygen, MV=mechanical ventilation.*

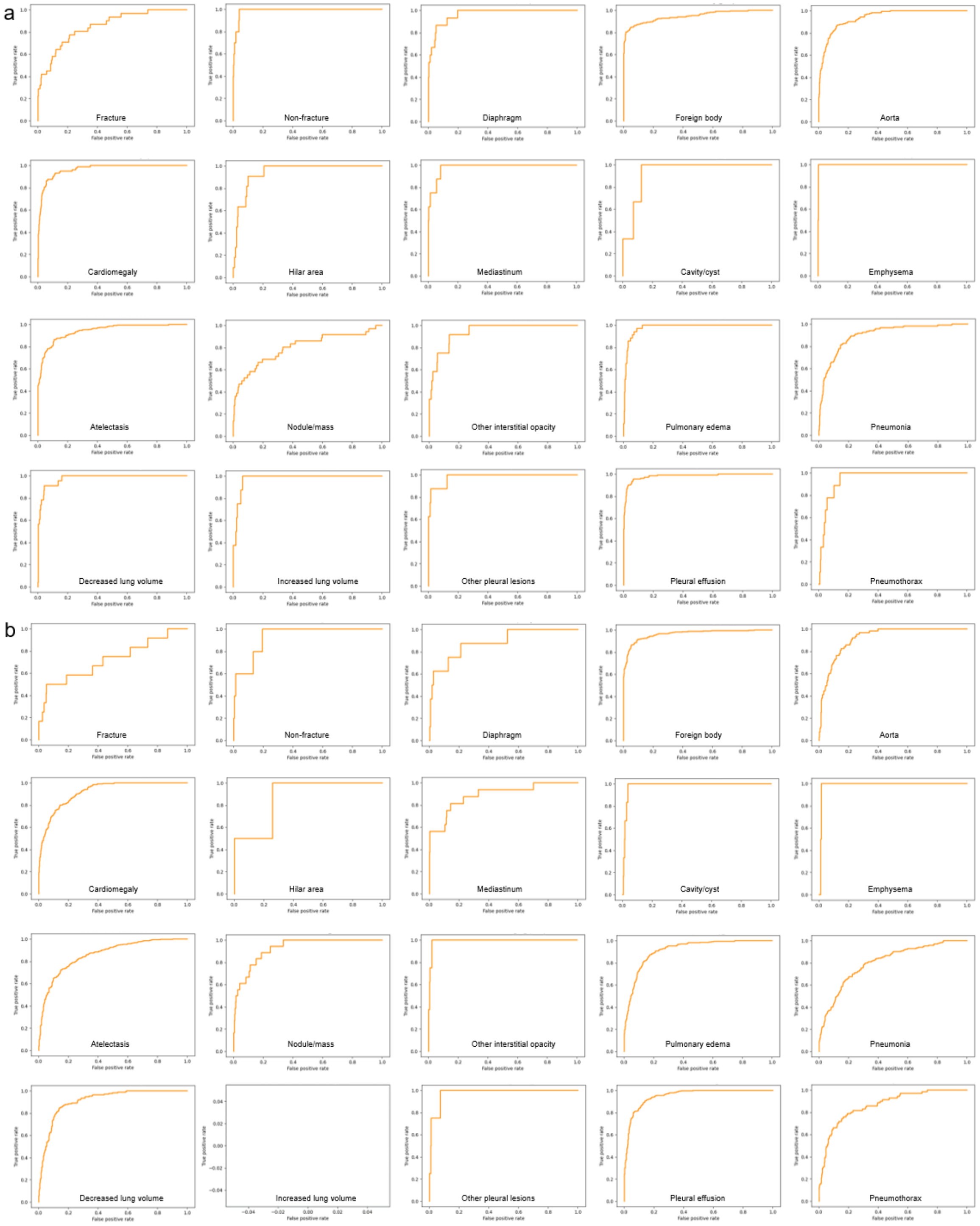

Supplementary Figure 1. ROCs for PA view (a) and AP view (b), derived from application of the final model to the test set. Non-fracture refers to non-fracture bone abnormality. Diaphragm, Aorta, Hilar area, and Mediastinum refer to diaphragmatic abnormality, aortic abnormality, hilar abnormality and mediastinal abnormality, respectively. As there were no cases with increased lung volume in the test set, AUROC for this label could not be calculated.

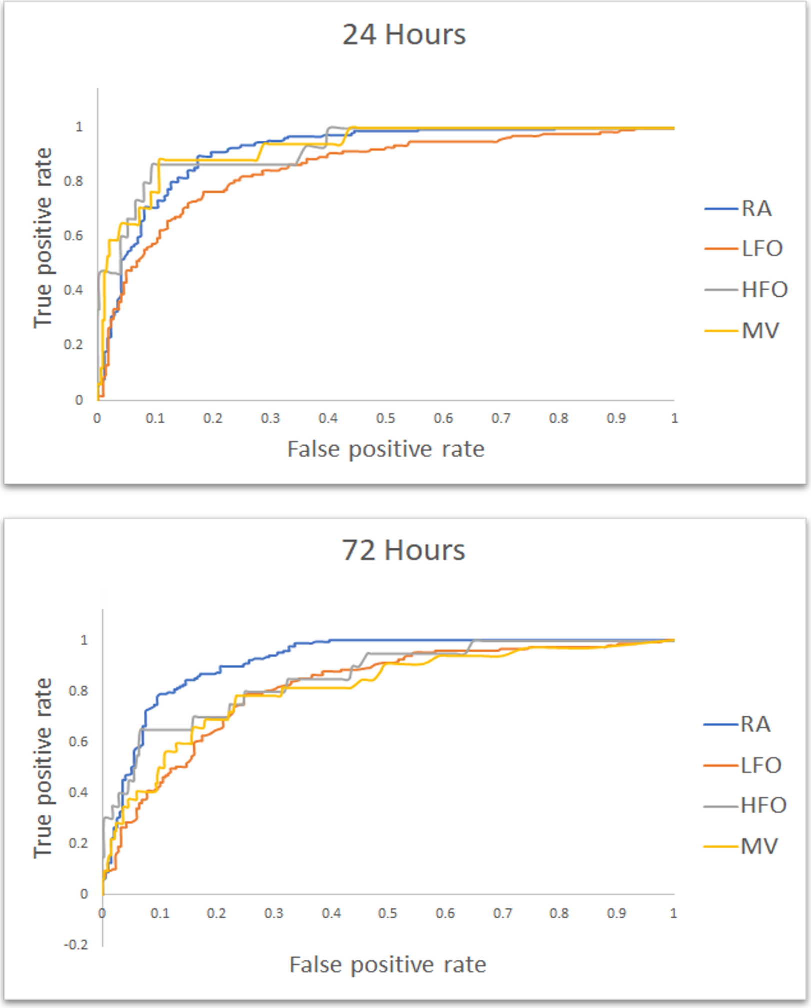

Supplementary Figure 2. ROCs for prediction of oxygen requirement at 24 and 72 hours from the time of ED admission in patients with COVID-19 infection using infection-associated radiographic features in combination with clinical information. *RA=room air, LFO=low flow oxygen, HFO=high flow oxygen, and MV=mechanical ventilation.*

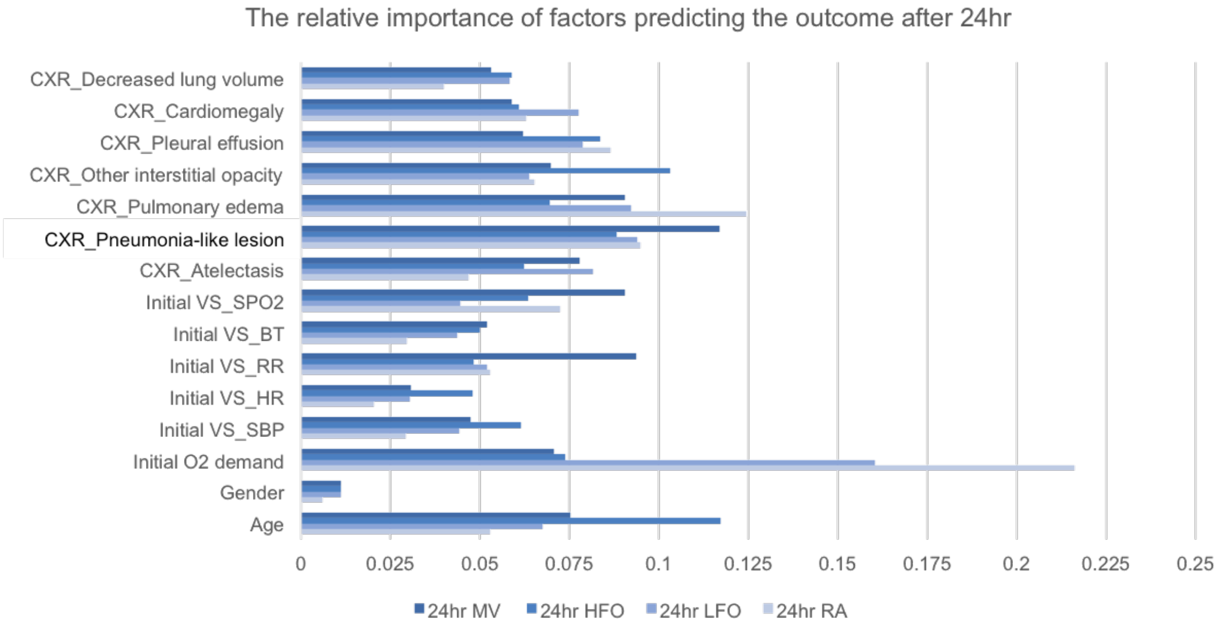

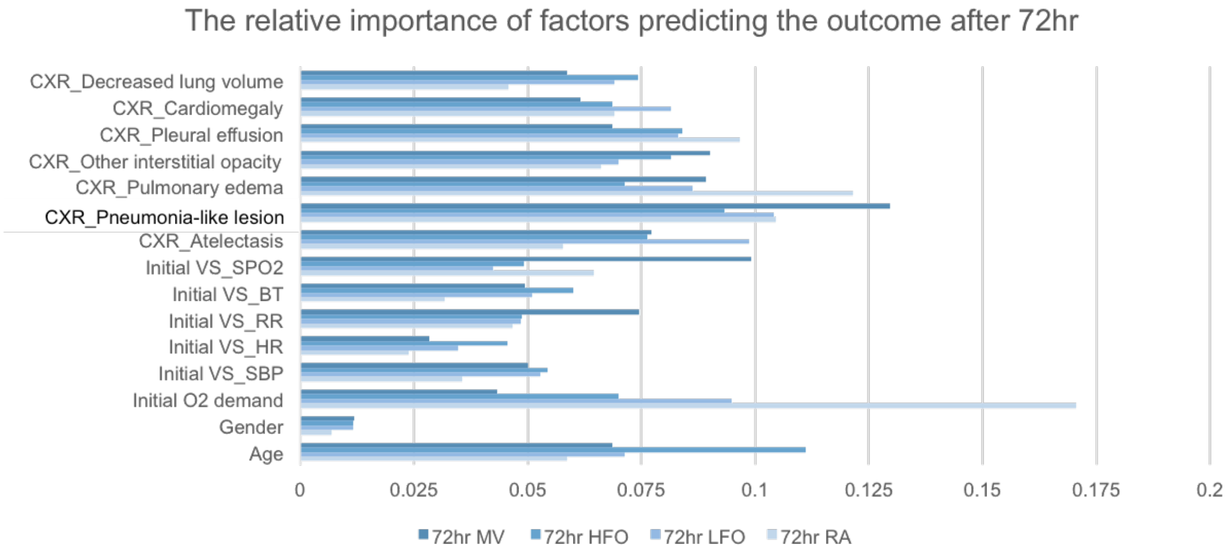

Supplementary Figure 3. Determinants of oxygen requirement at 24 and 72 hours from the time of ED admission in patients with COVID-19 infection.

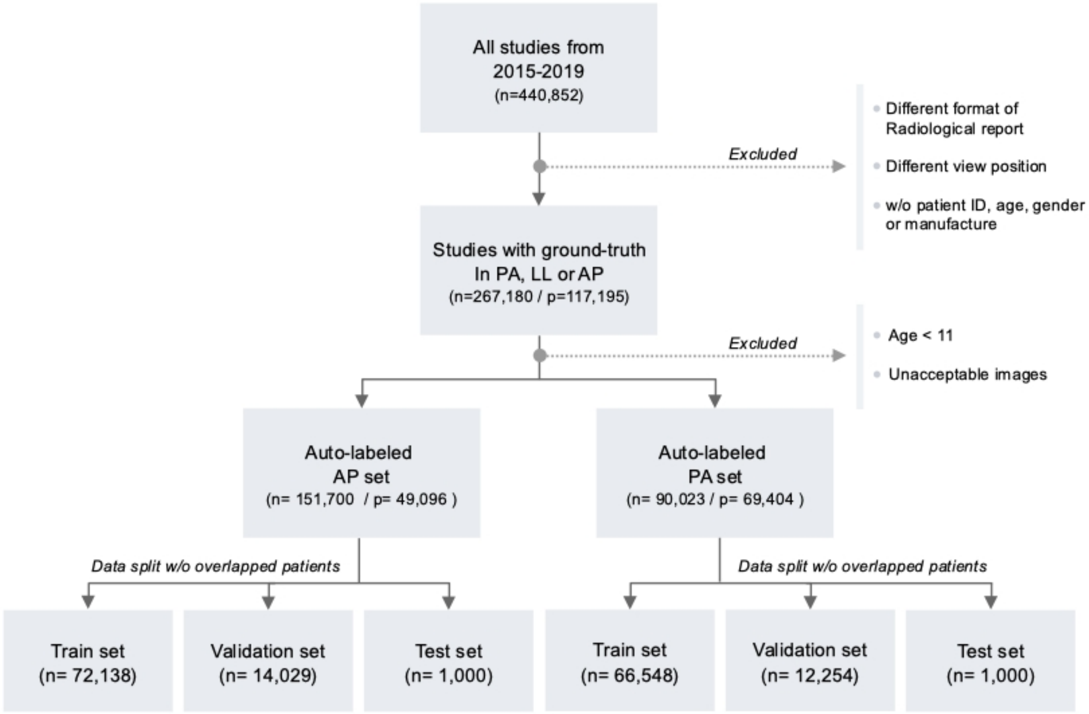

Supplementary Figure 4. Flowchart of dataset acquisition for xAI model development. AP and PA views were collected and mapped one-on-one to the annotations extracted by our NLP tool from the corresponding radiology reports. The AP and PA datasets were then divided into train, validation, and test sets without patient overlap. *Reproduced from Kim (2022)*[6].

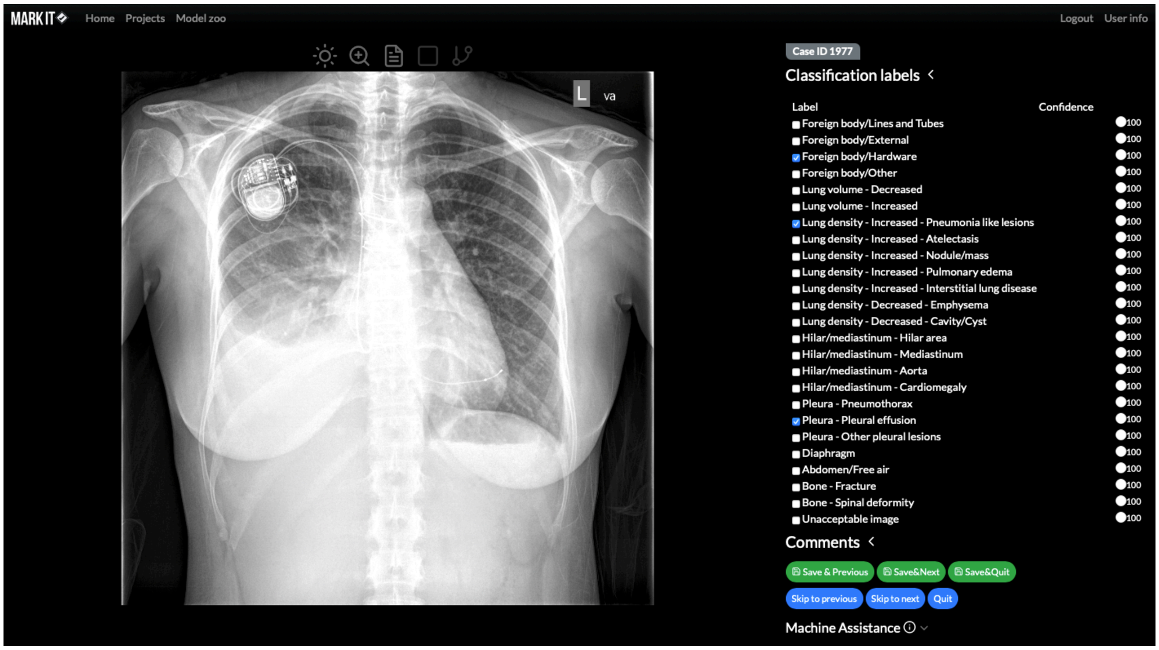

Supplementary Figure 5. A web-based annotation tool, Markit (https://markit.mgh.harvard.edu, MA, USA).

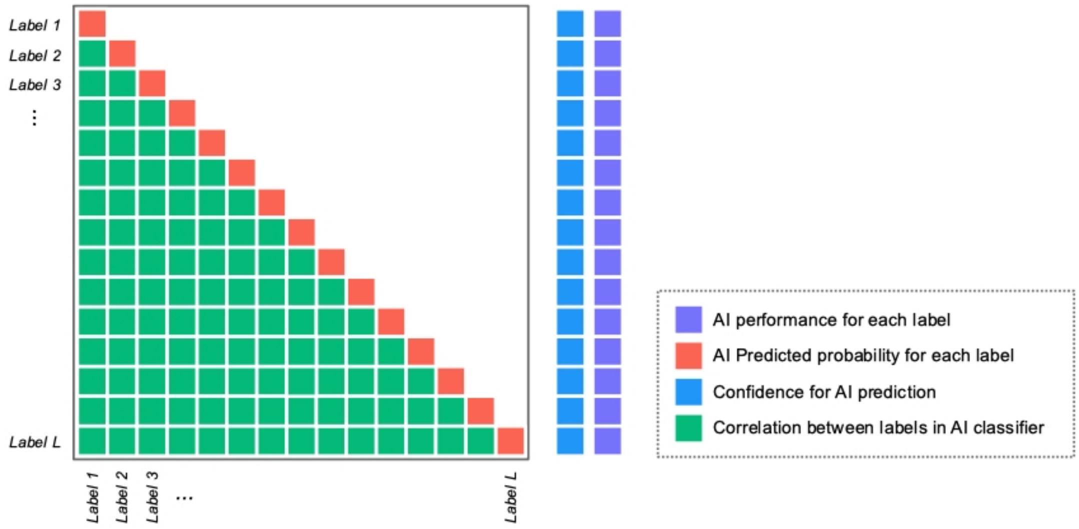

Supplementary Figure 6. AI scoreboard. The AI scoreboard is a diagram displaying quantitative metrics, such as prediction probabilities, calibrated classifier confidence, and correlation between labels.
